# The stem cell quiescence and niche signaling is disturbed in the hair follicle of the hairpoor mouse, an MUHH model mouse

**DOI:** 10.1186/s13287-022-02898-w

**Published:** 2022-05-26

**Authors:** Keonwoo Choi, Sang-Hee Park, Seo-Yeon Park, Sungjoo Kim Yoon

**Affiliations:** 1 Department of Biomedicine & Health Sciences, The Catholic University of Korea, Seoul, Republic of Korea; 2 Department of Medical Life Sciences, The Catholic University of Korea, 222 Banpo-daero, Seocho-ku, Seoul, 065-591 Republic of Korea

**Keywords:** Hairpoor mouse, MUHH, Hair follicle stem cell, Wnt signaling, Alopecia

## Abstract

**Background:**

Hair follicle stem cells (HFSC) play an essential role in the maintenance of hair homeostasis; during the hair cycle, HFSC remain quiescent for most of its duration. The hairpoor mouse (+ */Hr*^*Hp*^), an animal model of Marie-Unna hypotrichosis (MUHH), overexpresses hairless in the bulge, inner root sheath, and outer root sheath of HF and shows the same phenotype as in MUHH patients manifesting sparse hair with progression to alopecia with age. The aim of this study was to gain an understanding of the hair cycle and the status of HFSC during the hair cycle of the hairpoor mouse in order to delineate the pathogenesis of MUHH.

**Methods:**

H&E staining was performed in order to define the state of the hair follicle. FACS analysis and immunostaining were performed at the 1st and 2nd telogen stages for observation of the HFSC. A label retaining assay was performed to determine the quiescent state of hair follicles. qRT-PCR was performed to determine expression of factors involved in niche signaling and Wnt signaling.

**Results:**

We observed a drastic decrease in the number of hair follicles after the 1st telogen, followed by an intensified disturbance in the hair cycle with shorter anagen as well as 2nd telogen in the hairpoor mouse. A dramatic reduction in the number of CD34 expressing bulges as well as cells was observed at the telogen of the HFs, with prominent high proliferation of bulge cells, suggesting the loss of HFSC quiescence in the hairpoor mouse. The increased cell proliferation in HF was reiterated following the synchronization of the hair cycle, leading to acceleration of HF cycling. Reduced expression of *Fgf18* and *Bmp6,* the factors involved in HFSC quiescence, was observed in the HFSC niche of the hairpoor mouse. In addition, disturbed expression of Wnt signaling molecules including *Wnt7b*, *Wnt10b*, and *Sfrp1* was observed, which induced the telogen-to-anagen transition of HFs in the hairpoor mouse.

**Conclusions:**

These results indicate that the quiescent state of HFSC is not properly maintained in the hairpoor mouse, consequently leading HFs to the completely disarrayed hair cycle. These findings may provide an understanding of an underlying mechanism for development of alopecia with age in MUHH patients.

**Supplementary Information:**

The online version contains supplementary material available at 10.1186/s13287-022-02898-w.

## Background

Hair follicle stem cells (HFSC), located in the bulge of the hair follicle (HF), play an important role in the development of hair and maintenance of the HF cycle [1, 2]. During its lifetime, HF continuously undergoes a cycle of growth (anagen), degeneration (catagen), and rest phases (telogen) [3]. Regulation of HF cycling occurs through a precise interplay of several specific signaling activities [4].

Wnt signaling is a central regulator of hair morphogenesis and hair growth [5]. During the anagen phase, activation of Wnt signaling induces the HF cycle via the proliferation and differentiation of HFSC [6] and *Wnt7b* plays a role as a critical regulator of the HF cycle [7]. Increased expression of β-catenin, an intracellular signal transducer of the Wnt signaling pathway, occurs at the telogen–anagen transition and remains at the same level throughout the anagen phase [8, 9]. While Wnt signaling induces activation of HFSC proliferation, bone morphogenetic protein (BMP) signaling plays an important role in maintaining quiescence of HFSC [10, 11]. BMP is highly expressed in the HFSC niche during the telogen phase. Bone morphogenetic protein 6 (Bmp6) and fibroblast growth factor 18 (Fgf18), which have been shown to be essential in maintaining quiescence of HFSC, are highly expressed in Keratin 6 (K6) positive cells in the inner layer of the bulge [12]. Fgf18 conditional knockout mice display the shortened telogen phase [13], and similarly, overexpression of Noggin, a BMP antagonist, in mouse skin results in a shortened telogen phase and activation of HF growth [14]. Thus, homeostasis and cyclic activation of HFSC are controlled by the balance between Wnt, BMP, and Fgf18 signaling.

Hairless (*Hr*), a transcriptional corepressor, downregulates target genes in conjunction with the thyroid hormone receptor, retinoic acid receptor, and vitamin D receptor [15–17]. The *Hr* gene, which is expressed in the outer root sheath of HF, is involved in the development of HF through regulation of Wnt signaling [18, 19]. Associations of mutations in the *Hr* gene with hair loss disorders in humans and mice have been reported [20, 21]. Among them, mutations in the 5’UTR of *HR* are known to cause Marie-Unna hypotrichosis (MUHH), a genetic disorder of alopecia [22]. We recently reported on the mutant mouse known as “hairpoor” (*Hr*^*Hp*^) with T-to-A transversion substitution at position 403 in the second uORF of 5'UTR of the Hr gene (NM_021877) [23]. This mouse displays overexpression of HR and the characteristics of the human MUHH, which manifests sparse hair in early age and gradual hair loss with age [24]. Shortened hair follicles and abnormal differentiation of keratinocytes was observed in the hairpoor heterozygotes (+ /*Hr*^*Hp*^). In addition, unusually complicated Wnt signaling was demonstrated in this mouse [24, 25]. Although the characteristics of hairpoor mice have been described in these studies, the status of the HFSC in the HR overexpressing hairpoor mouse has not been fully examined.

In the current study, we examined the hair cycle of the hairpoor mouse and investigated the status of the HFSC. Our findings clearly demonstrated that the quiescence of HFSC was not maintained in the hairpoor mouse, exhibiting the shorter telogen phase and premature induction of anagen phase with proliferation of HFSC, consequently leading to a completely disarrayed HF cycle. These phenomena concurred with the downregulation of *Fgf18, Bmp6*, and *Sfrp1* and upregulation of *Wnt7b* and *Wnt10b* expression, presumably leading to failure of appropriate signaling activity in the HFSC niche.

## Methods

### Animals

Housing conditions of animals were as previously reported [21]. All procedures were conducted in accordance with the Laboratory Animals Welfare Act, the *Guide for the Care and Use of Laboratory Animals*, and the *Guidelines and Policies for Rodent Experiments* provided by the IACUC (Institutional Animal Care and Use Committee) in the School of Medicine, The Catholic University of Korea. Depilation was achieved by manual plucking of dorsal skin followed by wax strip depilation at 2nd telogen [3].

### Histology, immunofluorescence, and immunocytochemistry

Skin samples were taken at the 1st telogen (wild type: P21, hairpoor: P19-20), 2nd telogen (wild type: P49, hairpoor: P35), or indicated date. Tissues were fixed in 4% formaldehyde solution overnight at 4 °C and embedded in paraffin wax. Paraffin sections (6 μm) were prepared, and hematoxylin and eosin (H&E) staining was carried out following a standard protocol [24]. For immunofluorescence or immunohistochemical staining, antigen retrieval was performed in 10 mM sodium citrate (pH 6.0). Skin sections were blocked with 5% BSA in PBS for 1 h at room temperature and incubated with primary antibodies overnight at 4 °C. The primary antibodies used were anti-CD34 (1:200, Abcam, MA, USA), anti-K15 (1:200, Abcam, MA, USA), anti-NFATc1 (1:100, Santacruz, CA, USA), anti-LEF1 (1:100, Cell Signaling Technology, MA, USA), anti-SOX9 (1:100, Santacruz, CA, USA), anti-TCF4 (1:100, Cell Signaling Technology, MA, USA), anti-Ki67 (1:100, NeoMarkers, CA, USA), anti-BrdU (1:200, Abcam, MA, USA), anti-K6 (1:100, Abcam, MA, USA), anti-Bmp6 (1:100, Cell Signaling Technology, MA, USA), and anti-Fgf18 (1:100, Cell Signaling Technology, MA, USA). Alexa Fluor 488 or 594 goat anti-mouse, goat anti-rabbit, goat anti-rat secondary antibody (1:500, Invitrogen, MA, USA) were used for immunofluorescence. Counterstaining was done with DAPI. Fluorescence signal was observed with a fluorescent microscope (Olympus, Tokyo**,** Japan) or confocal microscope (Zeiss, Oberkochen, Germany). For immunohistochemical experiments, anti-mouse, anti-rabbit, and anti-rat HRP-conjugated antibodies (1:500, Santacruz, CA, USA) were utilized. Secondary antibodies were incubated for 2 h at room temperature. DAB chromogen was used for color reaction (Dako Glostrup, CA, USA), and counterstaining was done with hematoxylin. Image signal was observed with an optical microscope (Olympus, Tokyo**,** Japan).

### 5-bromodeoxy-uridine (BrdU) labeling

For label-retaining cell experiments, 10-day-old mice were injected with 5-bromodeoxy-uridine (BrdU, 50 μg/g) intraperitoneally (i.p) every 12 h for 48 h [26]. After a 60-day chase period, the dorsal skin was isolated. For proliferation analysis, BrdU (150 μg/g) was i.p injected to mice 1 h prior to kill at the indicated days.

### qRT-PCR

Total RNA was extracted from either dorsal skin or epidermis using Trizol reagent (Invitrogen, Carlsbad, CA, USA) following the manufacturer’s instructions. The single-stranded cDNAs were synthesized using the Prime Script 1st strand cDNA Synthesis kit (Takara, Tokyo, Japan) and used for real-time PCR using a CFX96 Touch™ Real-Time PCR Detection System (Bio-Rad Laboratories, Hercules, CA, USA). Primer sequences for each gene are listed in Additional file 1: Table S1.

### FACS analysis

Mouse epidermis were isolated from back skin of 1st and 2nd telogen mice as described previously [27]. Cells were labeled with anti-integrin α6 antibody conjugated to phycoerythrin (BD Bioscience, NJ, USA) and anti-CD34 conjugated to FITC (eBioscience, Waltham, MA, USA) for 30 min on ice. Cells were then analyzed using BD FACS CantoII sorter and FACSDiva™ software analysis system.

### Statistical analysis

*P *values were determined using Student’s t tests. Statistical significance was set at *p* < 0.05.

## Results

### Alteration in hair cycling of the hairpoor mouse

The hairpoor mouse exhibited increased proliferation and differentiation of epidermal cells [24] and premature catagen followed by the early telogen at the first hair cycle [25], indicating aberrant HF development. Because the abnormal hair loss with aging in hairpoor mice was similar to that of MUHH patients, we hypothesized that HFSC might be affected in this disorder leading to an abnormal HF cycle, resulting in acceleration of hair loss.

**Fig. 1:**
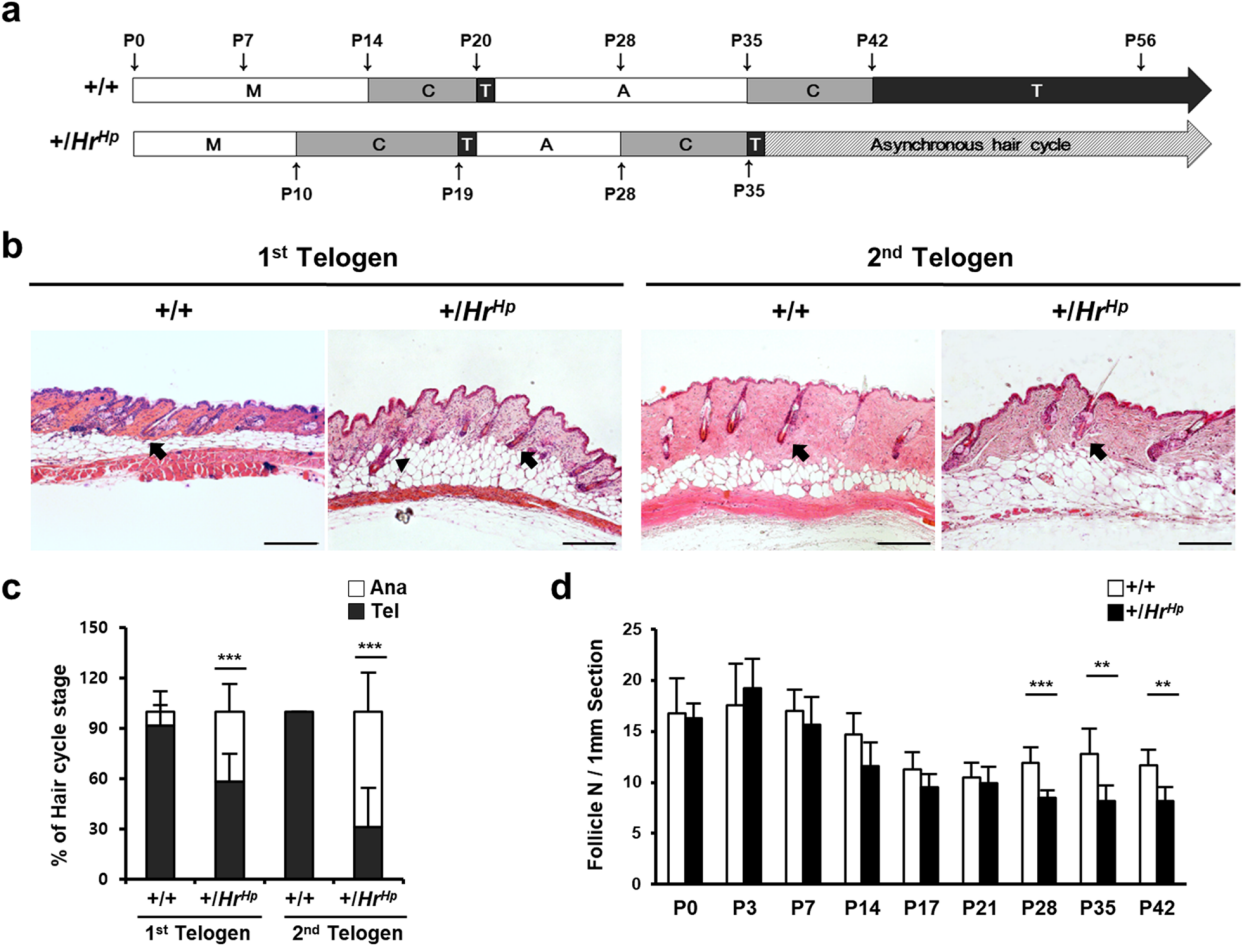
Histological analysis of hair cycle in hairpoor mouse. **a** Schematic presentation of the hair cycle. Morphogenesis, M; catagen, C; telogen, T; anagen, A. **b** Hematoxylin and eosin (H&E) stain of the 1st and 2nd telogen HFs in the hairpoor mouse and the wild-type mouse (+ */* +). Arrow indicates telogen follicle and arrowhead anagen follicle. **c** Percentage of HFs of each stage of the hair cycle at the indicated telogen phase. Mouse number *n* ≥ 5 per genotype. Number of HFs counted in each mouse > 20. **d** Number of hair follicles per 1 mm section of back skin. Data are mean ± SD. *n* ≥ 5 mice per genotype/group. Scale bar = 200 μm. Data are mean ± SD. *P < 0.05, ***P < 0.001

To examine this hypothesis, we first observed and compared the hair cycle between hairpoor and littermate wild-type mice, including the second telogen stage (Additional file 2: Fig. S1 and Fig. 1a). Results of H&E staining showed a much shortened anagen stage following the premature catagen and earlier onset of the 1st telogen in the hairpoor mouse compared to that of the wild-type mouse. At P19-20, the HF of + */Hr*^*Hp*^ was at the 1^st^ telogen stage, while HF of the wild-type mouse was still at the 1st catagen, showing the earlier onset of telogen in the hairpoor mouse. Due to the shortened cycle, the onset of the 2nd telogen was much earlier and its duration was remarkably shorter in the hairpoor mouse compared to the wild-type mouse. The 2nd telogen started at P35 and lasted for approximately one day (P35-P36) in hairpoor HF, while it started at P42 and lasted for more than 2 weeks in wild-type HF (Fig. 1a).

Among the stage of telogen HFs of the hairpoor mouse, the clear morphological abnormality was not observed compared to the wild-type HF as shown in Fig. 1b. However, stages of the hairpoor mouse differed significantly from that of the wild-type mouse. At the 1st telogen, the proportion of telogen HF was 91.5% in the wild-type mouse, whereas it was only 58.3% in the hairpoor mouse. In the case of the 2nd telogen, all the wild-type HF was at telogen phase, but only about 30% of the hairpoor HF were at telogen phase and 70% were already into the anagen phase (Fig. 1c). Following the short 2nd telogen, the HFs were a mixture of mostly anagen, a few catagen, and telogen HFs in the hairpoor mouse (data not shown).

Of particular interest, no significant difference in the number of HFs was observed between the wild-type and the hairpoor mouse until P21. However, at P28, the number of HFs in + */Hr*^*Hp*^ was drastically lower than that of the wild-type mouse and the difference was maintained throughout the observed period (Fig. 1d), suggesting that the capability of HFs for regeneration appeared to have been affected by the first telogen and those defects persisted in the hairpoor mouse. These results clearly indicated that the transition of the stages of the HF cycle was abnormal in the hairpoor mouse and raised a possibility of the impairment of HFSC that controls the HF cycle.

### ***Decrease in the number of CD34***^+^***HFSC in hairpoor mice***

**Fig. 2:**
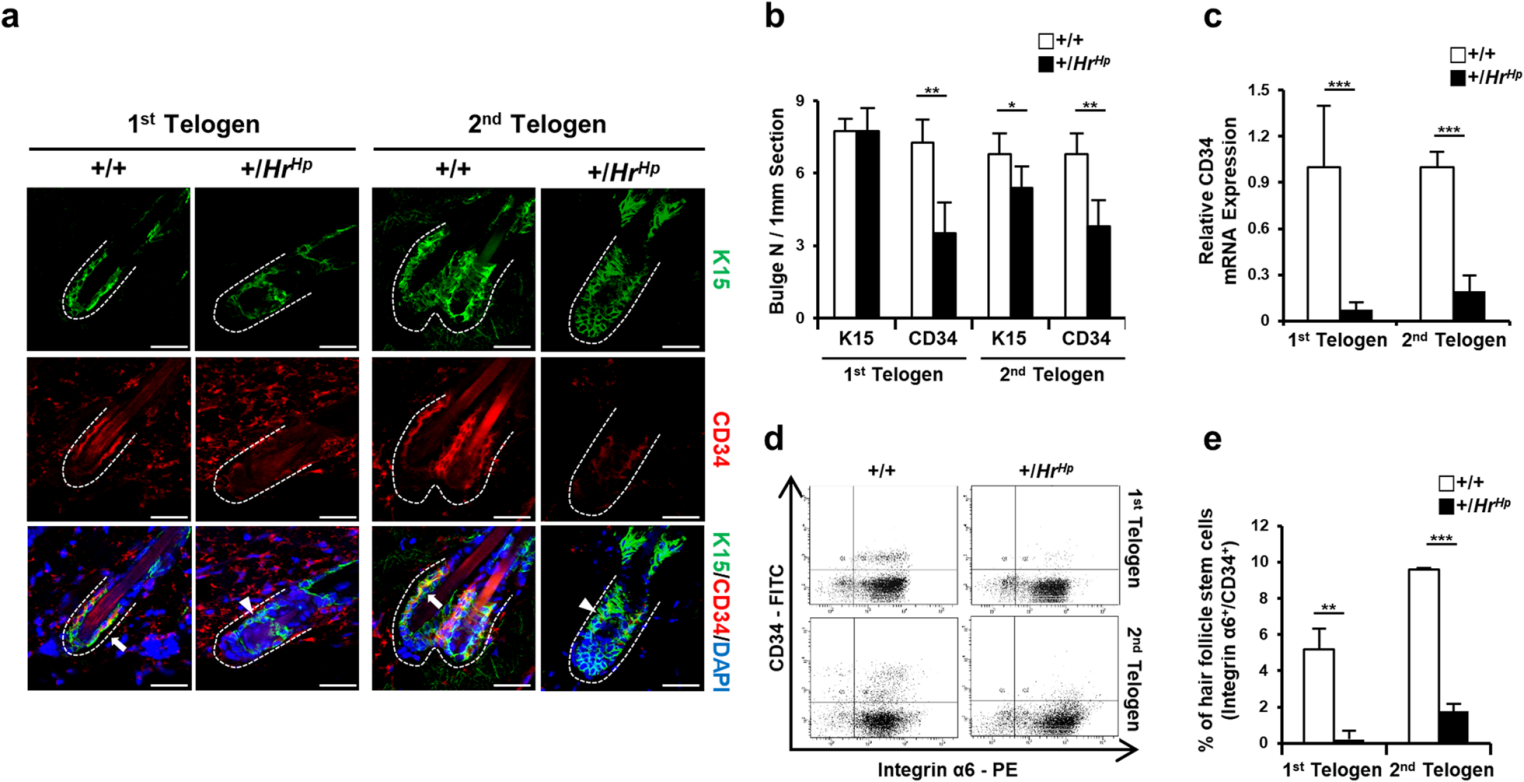
Decreased expression of CD34, a hair follicle stem cell (HFSC) marker, in the hairpoor mouse. **a** Immunofluorescence analyses of K15 (green) and CD34 (red) in wild-type and hairpoor mice at the telogen phases. The arrow indicates K15 and CD34 doubly positive bulges and the arrowhead K15 only positive bulges. Nuclei were counterstained with DAPI. Bulge, Bu; scale bar = 50 μm. **b** Number of K15 or CD34 positive HFs per 1 mm section of the back skin of wild-type and hairpoor mouse (mouse number *n* ≥ 4 per genotype). Data are mean ± SD. *P < 0.05, **P < 0.01. **c** qRT-PCR analysis of *Cd34* in the hairpoor and the wild-type mouse. (Mouse number *n* ≥ 3 per genotype). Expression level of *Cd34* was normalized against that of *Gapdh*, and relative expression level was determined using one of the wild-type levels as reference. Data are mean ± SD, ***P < 0.001. **d** Representative result of the flow cytometry analysis at telogen phases. **e** Proportion of the ITGA6^+^/CD34^+^ HFSC in the epidermal cells prepared from the back skins of the hairpoor and wild-type mouse. Data are mean ± SD. *n* = 3 mice per genotype. **P < 0.01, ***P < 0.001

In order to dissect the abnormal hair cycle in the hairpoor mouse, we attempted to determine whether there were any changes in HFSC. First, the number of bulges expressing K15, a bulge marker, and CD34, a HFSC marker [28], was determined at telogen stages using immunohistochemistry. At both telogen stages, all K15^+^ bulges had similar intensities for both wild-type and hairpoor mice (Fig. 2a). In contrast to K15, CD34 expression displayed a different pattern between two lines. Almost all the K15^+^ bulges were CD34^+^ at the first telogen (7.25/7.5) and the second telogen (6.8/6.8) stages of the wild-type HFs. In contrast, on average, only 45% (3.5/7.75) and 70% (3.8/5.4) of the K15^+^ bulges showed CD34^+^ expression at the first and second telogen of the hairpoor HFs, respectively. In determining the number of CD34^+^ bulges, the bulge showing a detectable expression level of CD34 was regarded as a CD34^+^ bulge (Fig. 2b). In addition to the reduced number of CD34^+^ bulges, much weaker expression was observed in the CD34^+^ bulges, of hairpoor HFs compared to those of wild-type HFs (Fig. 2a, b). Of particular interest, a higher proportion of CD34^+^/K15^+^ bulges was observed at the 2nd telogen than at the 1st telogen of hairpoor HFs, although the expression level was much lower than that of wild-type HF.

qRT-PCR was performed to determine the expression level of *Cd34* mRNA in order to assess the extent of the reduced expression of CD34 in hairpoor HFs. A significant reduction in the relative expression level of *Cd34* mRNA was observed in the epidermis of hairpoor compared to that of wild type (Fig. 2c), with less than 20% of that of the wild-type mouse for both telogen stages.

Next, using FACS analysis, we determined the proportion of HFSC (ITGA6^+^/CD34^+^) in the bulge at both telogen stages (Fig. 2d, e). In wild-type mice, ITGA6^+^/CD34^+^ cells composed 5.2% and 9.6% of the bulge cells at the 1^st^ and 2nd telogen stages, respectively. In contrast, the bulges in hairpoor mice contained only 0.2% and 1.8% ITGA6^+^/CD34^+^ cells of the bulge cells at the 1^st^ and the 2nd telogen stages, respectively. This result clearly indicates that the number of HFSC expressing CD34 was significantly reduced in the hairpoor mouse. Taken together, significantly fewer HFSC present in the HFs were observed in the hairpoor mouse compared to the wild type, suggesting aberrance in the maintenance of HFSC in the hairpoor mouse.

### Loss of HFSC quiescence and increased cell proliferation in the hairpoor HF

**Fig. 3:**
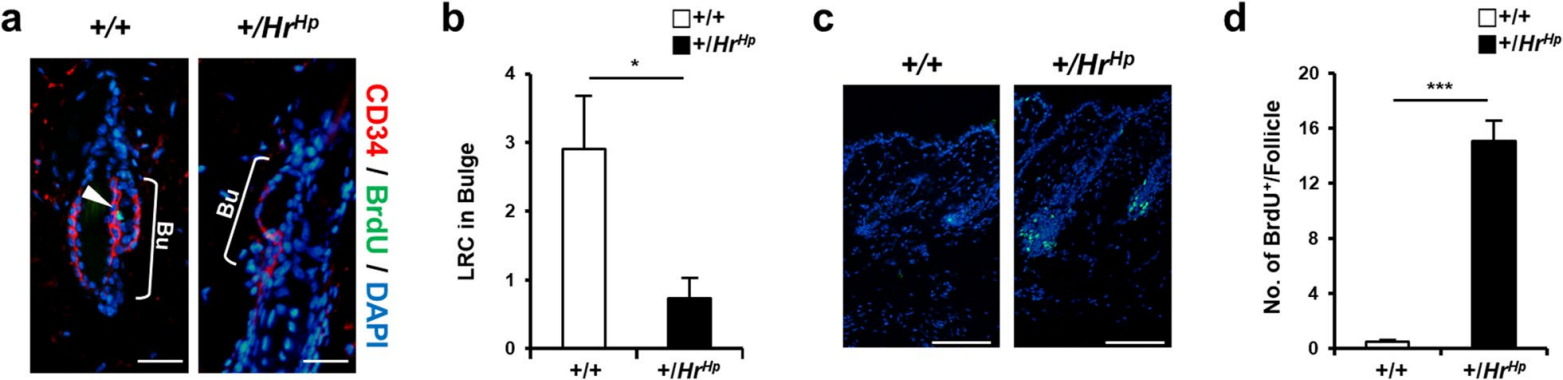
Hairpoor mouse displays the loss of label-retaining HFSC and increased cell proliferation in HF. **a** Immunofluorescence staining of BrdU-LRC in the CD34^+^ bulge at P70. White arrowhead indicates LRC in bulge. Bulge, Bu; scale bar = 25 μm. **b** Average number of LRC in bulge at P70. Data are mean ± SD. *P < 0.05. Mouse number *n* = 3 per genotype. Observed follicle number per each mouse *n* = 10. **c** Immunofluorescence staining of BrdU (1 h chase) at anagen II HFs (the wild-type mouse at P23 and the hairpoor mouse at P21). Scale bar = 100 μm. **a,c** Nuclei were counterstained with DAPI. **d** Quantification of BrdU positive cells in follicle. Data are mean ± SD. **P < 0.01, ***P < 0.001. *n* = 3 per genotype. Number of HFs counted in each mouse ≥ 10

In order to determine whether the quiescence of HFSC was disturbed in the hairpoor mouse, we first determined the number of label-retaining cells (LRC) in HF using the 5-bromodeoxy-uridine (BrdU) incorporation method at 60 days post an injection of BrdU at P10. Less than one BrdU^+^ LRC was detected in the bulge of hairpoor HF, while the wild-type HF contained three BrdU^+^ LRCs on average (Fig. 3a, b). Cell proliferation in HFs was then determined in order to delineate whether HFSC lost quiescence. Incorporation of BrdU in cells 1 h after BrdU injection was measured for assessment of cell proliferation. The experiments were performed at the anagen I–II period for both wild-type and hairpoor mice and revealed that the hairpoor mouse exhibited 15 BrdU^+^ cells/HF on average, while the wild-type mouse showed 0.48 BrdU^+^ cells/HF (Fig. 3c, d), showing excessive cell proliferation in hairpoor HFs.

**Fig. 4:**
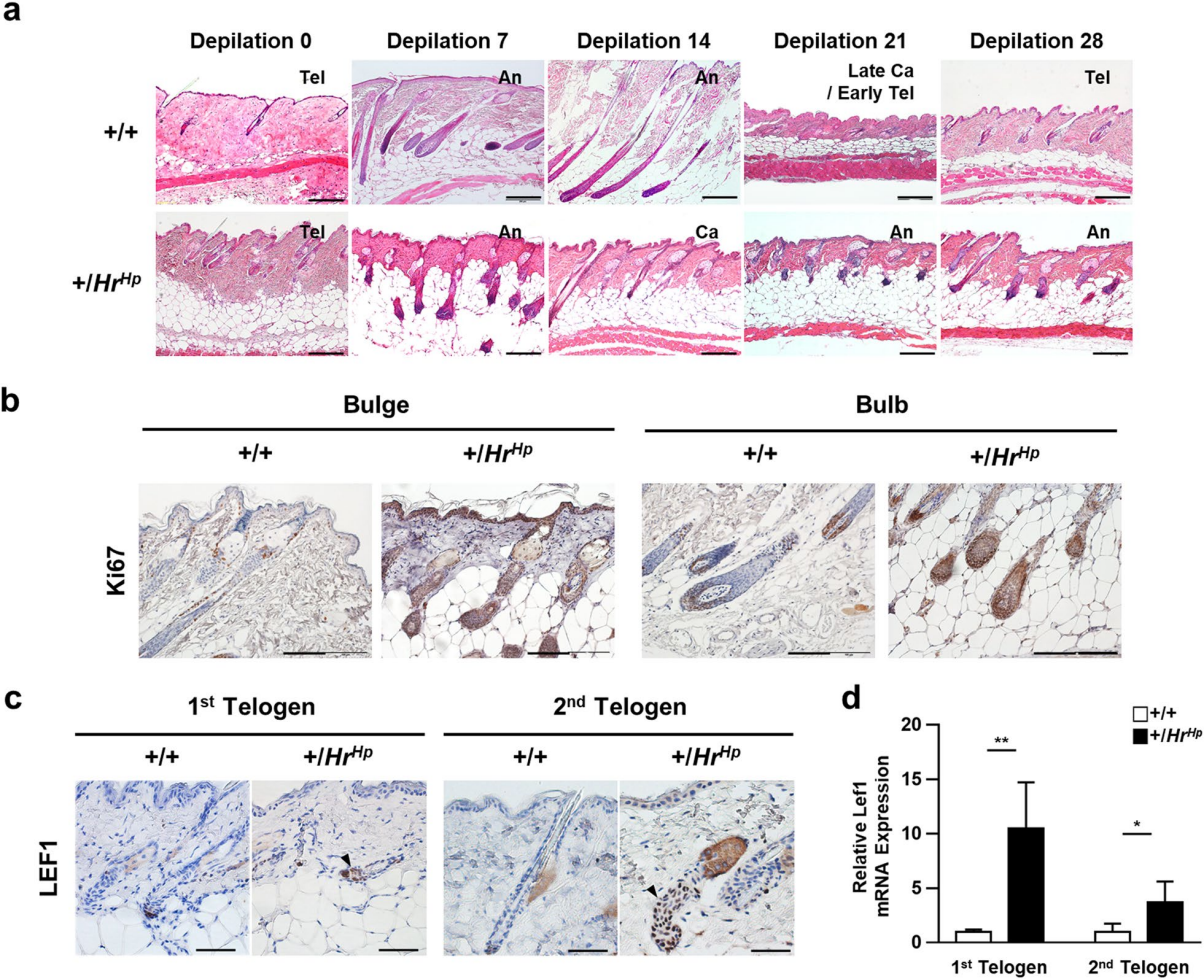
The hair cycle of the hairpoor mouse after depilation is impaired. **a** Histological analysis of hair cycle after depilation using H&E staining. Telogen, Tel; anagen, An; catagen, Ca; scale bar = 200 μm. **b** Immunohistochemical analysis of Ki67 in bulge and bulb using 6-μm-thick sections of the wild-type and the hairpoor mouse at 7 days after depilation. Scale bar = 100 μm. **c** Immunohistochemical staining of LEF1 at 1st and 2nd telogen phase. Arrowhead indicates positive staining. Scale bar = 50 μm. **d** qRT-PCR analysis of *Lef1* in the hairpoor and the wild-type mouse. Expression of *Lef1* was normalized against that of *Gapdh*, and the relative expression level was determined using one of the wild-type levels as reference. *n* = 4 per genotype. Data are mean ± SD. *P < *0.05*, ***P < 0.001

To further characterize HFSC in the hairpoor mouse, a histological study was performed on skin sections prepared after synchronization of HF cycling via depilation at the 2nd telogen phase. As shown in Fig. 4a, the HF cycle of the hairpoor mouse was much shorter than that of the wild-type mouse. At 14 days post-depilation, while the wild-type HFs were still in the full anagen phase, the hairpoor HFs were into the catagen phase (Fig. 4a). This deviation in the hair cycle of the hairpoor mouse was more prominent in subsequent days. Specifically, at 21 days post-depilation, the hairpoor HFs were in the anagen phase again, whereas the wild-type HFs were at the late catagen/early telogen phase, indicating that the telogen stage of the hairpoor HFs passed between 14 and 21 days post-depilation. In contrast, the wild-type HFs began the telogen stage at 21 days and remained in the same stage at 28 days post-depilation, while the hairpoor HFs were in anagen stage again.

Cell proliferation in HFs was examined at 7 days after depilation when both the wild-type and the hairpoor HFs were in anagen phase. Using immunohistochemical detection of Ki67, there is a higher proliferation of the cells in the bulge and bulb of the hairpoor HFs compared to those of wild-type HFs (Fig. 4b). In addition, expression of Lymphoid enhancer-binding factor 1 (LEF1), transcription activator in the presence of ß-catenin, was observed at both the bulges of 1st and 2nd telogen staged HFs of the hairpoor mouse (Fig. 4c, d). Taken together, quiescence could not be maintained by HFSC in the hairpoor mouse, resulting in proliferation of cells in HF.

### Expression of HFSC markers in hairpoor mice

**Fig. 5:**
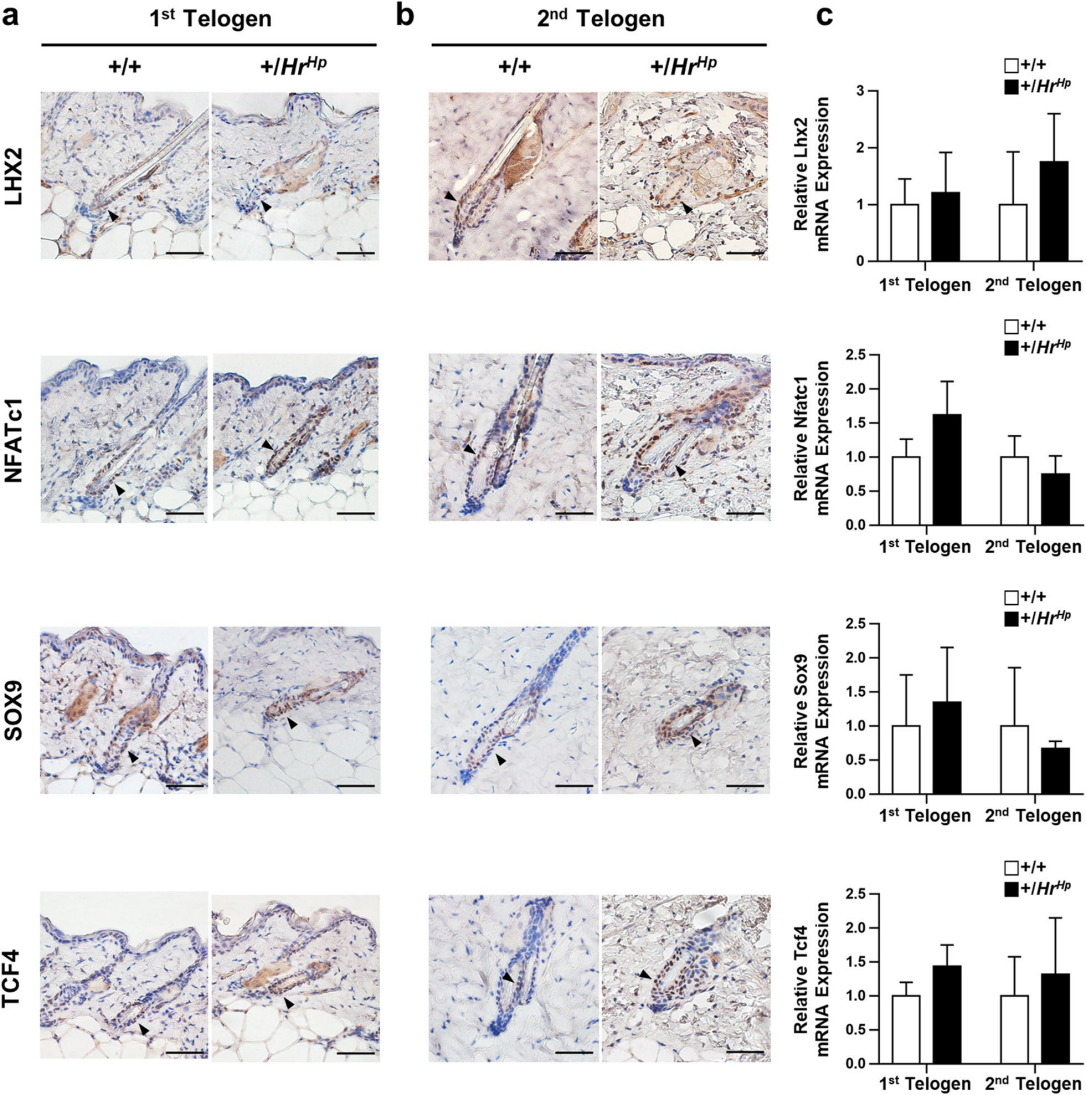
Expression of the HFSC quiescence markers in the hairpoor mouse. **a**, **b** Immunohistochemical staining of the bulge HFSC markers LHX2, NFATc1, SOX9, and TCF4, at 1st (a) and 2nd (b) telogen. Arrowhead indicates expression of each marker in the bulge (dark brown). All scale bar = 50 μm. **c** Relative expression level of HFSC markers (Lhx2, Nfatc1, Sox9, and Tcf4) in the stage of telogen total skin of the hairpoor and wild-type mouse determined by qRT-PCR analyses. mRNA expression level was normalized against that of *Gapdh*, and relative expression level was determined using one of the wild-type levels as reference. *n* = 4 per genotype. Data are mean ± SD

Expression of various HFSC markers including LIM homeobox 2 (LHX2), SRY-box 9 (SOX9), nuclear factor of activated T cell 1 (NFATc1), and transcription factor 4 (TCF4) [29–33] was observed using immunohistochemistry and qRT-PCR in order to dissect the status of the HFSC of hairpoor mice. LHX2, which is expressed in the bulge, plays an essential role in morphogenesis of HF as well as in maintenance of quiescence of HFSC. SOX9, which is known to have an association with Shh signaling, is expressed in the bulge and outer root sheath. As shown in Fig. 5a, b, no significant difference in expression of LHX2 and SOX9 was observed in the 1st telogen and 2nd telogen phases of HF of both the wild-type and the hairpoor mouse HFs. Expression of other bulge markers, NFATc1 and TCF4, was also detected in the junctional zone and interfollicular epidermis, and the bulges, respectively. TCF4, which has been shown to suppress HFSC quiescence and thus inhibit transition of HFs from telogen to anagen, was also expressed in the bulge. As with LHX2 and SOX9, no noticeable difference in expression of NFATc1 and TCF4 was observed between hairpoor and wild-type HFs (Fig. 5c). Thus, no difference in expression of all the HFSC markers tested in this study with exception of CD34 was observed between the hairpoor and the wild type.

### Disruption of HFSC quiescence signaling in the hairpoor mouse

**Fig. 6:**
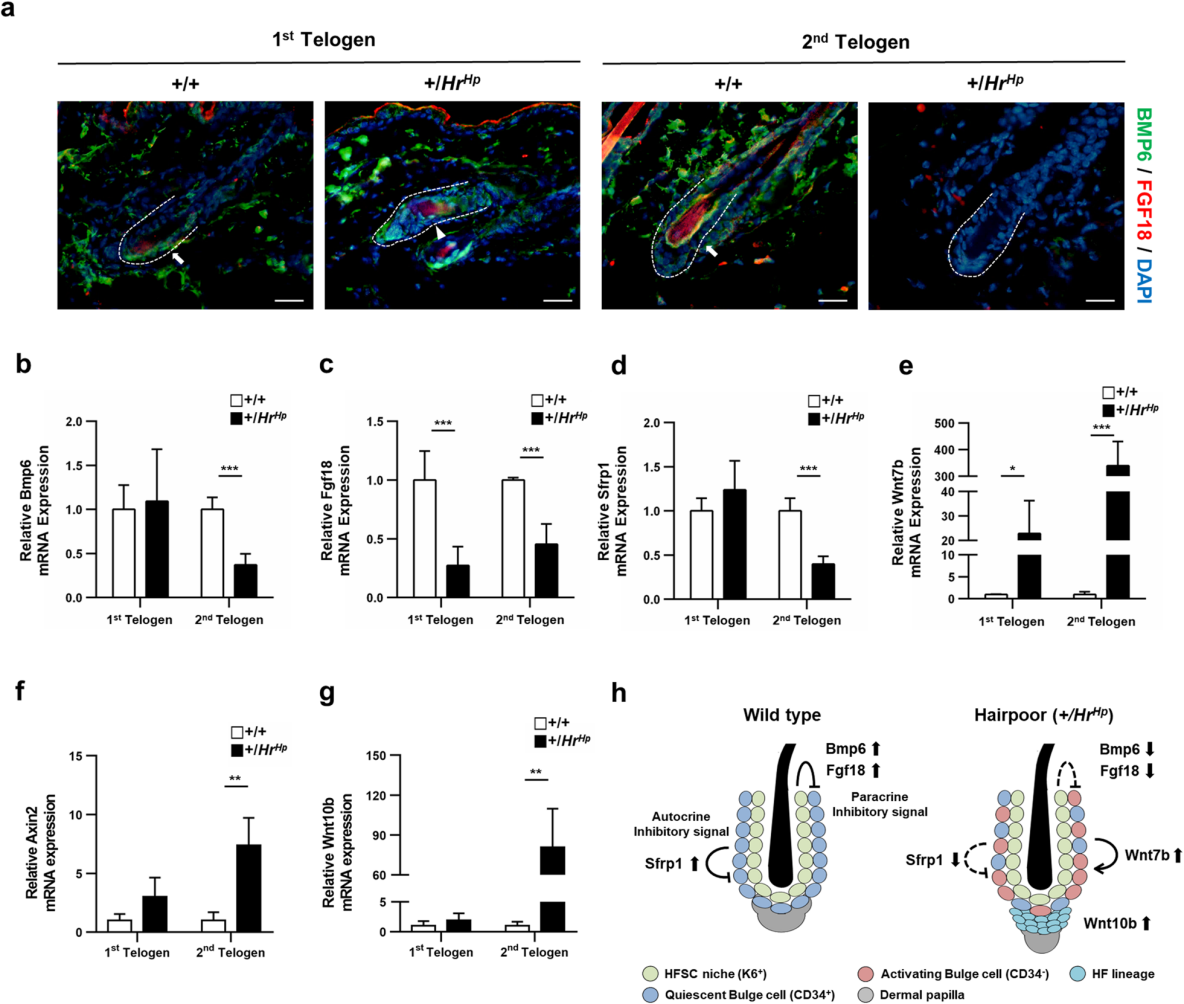
Expression of *Bmp6, Fgf18*, and Wnt signaling molecules is disturbed in hairpoor mouse. **a** Immunohistochemical staining of Bmp6 (green) and Fgf18 (red) in telogen phage. The arrow indicates Bmp6 and Fgf18 doubly positive cells and the arrowhead Bmp6 only positive cells. Nuclei were counterstained with DAPI. Bulge. Scale bar = 50 μm. **b**, **c** Expression level of *Bmp6* and *Fgf18* of the epidermis at the 1st and 2nd telogen phase of the hairpoor and the wild-type mouse determined by qRT-PCR analyses. **d,e,f,g** Expression level of the Wnt signaling molecules (*Sfrp1, Axin2, Wnt7b, Wnt10b*) in the stage of telogen total skin of the hairpoor and wild-type mouse determined by qRT-PCR analyses. **h** Schematic of the telogen status of each mouse. Expression level was normalized against that of *Gapdh*, and the relative expression level was determined using those of the wild-type levels as reference. *n* ≥ 3 per genotype. Data are mean ± SD. *P < *0.05*, ***P < 0.001

Next, we addressed the question of whether HFSC niche signaling was involved in the abnormal hair cycling of the hairpoor mouse. Using IHC, we observed the expression of K6, a HFSC niche marker; according to the results, all the K15^+^ bulges showed K6^+^ in the hairpoor HFs as the same, in the wild type (Additional file 3: Fig. S2). Because regulation of the quiescence of HFSC through expression of *Bmp6* and *Fgf18* by K6^+^ has been demonstrated, we also observed expression of *Bmp6* and *Fgf18* in the epidermis of mice [12]. The pattern of expression of *Bmp6* differed from that of *Fgf18*. Significantly decreased expression of *Bmp6* was observed only in the 2nd telogen phase in hairpoor HFs compared to that of wild-type HFs. In contrast to *Bmp6,* significantly decreased expression of *Fgf18* was observed in both the 1st and 2nd telogen phases in the hairpoor HFs compared to the wild-type ones (Fig. 6a, b and c). These findings demonstrate that reduced expression of *Fgf18* and *Bmp6* concurred with disturbance of HFSC quiescence in hairpoor mice, suggesting that the lack of expression of the quiescence maintaining regulators may cause disarrayed HF cycling. Next, we attempted to determine whether the HF in the telogen phase of the hairpoor mouse was in the competent telogen state or the telogen–anagen transition state [4] by determining the expression of Wnt signaling molecules in the skin. Expression of *Sfrp1*, a Wnt inhibitor, was reduced in the 2nd telogen of the hairpoor HFs to 40% of that of the wild type (Fig. 6d), and expression of *Wnt7b* was increased in both 1st and 2nd telogen in the hairpoor compared to the wild type (Fig. 6e). In addition, remarkably increased expression of *Axin2* and *Wnt10b* mRNA was observed in the 2nd telogen of the hairpoor mouse compared to the wild-type mouse (Fig. 6f, g). These results were the same even when normalized to *Sdha* (Additional file 4: Fig. S3). These observations suggest that overexpression of HR in the hairpoor mouse led to the reduction of HFSC quiescence signaling molecules in the HFSC niche and concomitantly activated the Wnt signaling pathway, resulting in transition of the hairpoor HFs from telogen-to-telogen–anagen transition state.

## Discussion

HR is expressed in HF and regulates downstream gene expression as a transcriptional cofactor [17, 34]. In skin, HR regulates the HF cycle by regulating Wise expression through involvement in Wnt signaling [34]. However, association of HR with HFSC has not been examined. Here, we report that overexpression of HR resulted in complete disruption of the HF cycle which may be caused by abnormal expression of molecules involved in regulation of HFSC in the HF niche using the hairpoor mouse.

The hairpoor mouse (+ */Hr*^*Hp*^) is a human MUHH disease model, and this heterozygote displays scarce hair during young age and then gradual progression to baldness with aging as manifested by MUHH patients [23, 24]. We previously reported that the length of HF was shorter, with a premature catagen stage followed by earlier onset of telogen stage in the first cycle of the hairpoor mouse compared to that of the wild-type mouse [25]. This abnormal hair cycle was further explored in the current study and found that the abnormality of the early HF cycle is accentuated in the 2nd cycle. The 2nd cycle of the hairpoor HF displayed a much shorter anagen stage and a lack of stable telogen stage which was composed of a mixed population of anagen and stage of telogen HFs, whereas the wild-type HFs showed the stable and long 2nd telogen stage with all HFs at telogen phase (Fig. 1, Additional file 2: Fig. S1). In addition, the reduced numbers of HFs were observed in the 4-week-old mice, which persisted until at least P56 of hairpoor mice. This phenomenon continued, and the decrease in number of HFs became greater in mice older than 1 year (data not shown). These results indicate that overexpression of *Hr* disturbed not only the first cycle but also the following cycles, suggesting involvement of *Hr* in the status of HFSC.

Expression of stem cell-specific markers provides valuable information in determination of the status of stem cells. Of the HFSC associated markers examined in this study, all the markers including NFATc1, SOX9, LHX2, and TCF4 with exception of CD34 expressed in all the K15^+^ bulges of hairpoor HFs. Expression of these HFSC markers has been shown to determine the quiescent state or in transition from telogen to anagen in HF [12, 28, 30–32, 35]. Loss of SOX9 was reported to cause failure in maintaining HFSC quiescence in conjunction with reduced expression of CD34 [36]. Of particular interest, reduced expression of CD34 was observed for the HFSC of the hairpoor mouse, while SOX9 expression was not affected compared to the wild-type HFSC. In addition, expression of LHX2, which occurs in both embryonic hair placodes and postnatal HFSC [30], has no significant difference in the hairpoor mouse. Surprisingly, only CD34 showed dramatically reduced expression at the 1^st^ telogen HFs in the hairpoor mouse. Expression of CD34, the most widely used HFSC marker, is known to occur in the bulges including 1^st^ telogen stage [37, 38], and the importance of its expression in the integrity of HFSC has been demonstrated [39, 40]. Thus, reduction in expression of CD34 in the bulges of 1^st^ telogen staged HFs strongly suggested the loss of the quiescence of the HFSC. The loss of HFSC quiescence was further supported by the accelerated cell proliferation in hairpoor HFs and recapitulation of the same results as shown in the disarrayed hair cycle in the depilation experiment.

Due to the lack of differences in expression of HFSC markers other than CD34, we further investigated expression of the niche markers. While no change was observed in the expression of K6, a HFSC niche marker, expression of *Bmp6* and *Fgf18*, which are known to maintain telogen state [41, 42], was found to show a dramatic reduction in the hairpoor mouse. Of particular interest, at the 1st telogen, the expression level of *Fgf18* was distinctly reduced, while *Bmp6* was not. *Foxp1* was recently shown to maintain HFSC quiescence through *Fgf18* without altering expression of *Bmp6* [43]. Our results clearly provide the evidence that *Bmp6* and *Fgf18* expressed in the K6^+^ inner bulge play different roles in maintaining HFSC quiescence. The relationship between *Hr* and *Foxp1* remains to be explored.

During the telogen, expression of *Sfrp1*, a Wnt inhibitor, was observed in both inner and outer bulges and was in balance with Wnt7b expression in the niche cells with *Axin2* expression in maintaining HFSC quiescence [44]. In the hairpoor mouse, markedly reduced expression of *Sfrp1* was observed in the 2nd telogen. We have shown that expression of HR resulted in suppressed expression of *Sfrp1* mRNA [45]. Thus, presumably overexpression of HR contributes in part to loss of HFSC quiescence via downregulation of *Sfrp1* expression in the hairpoor mouse. Activation of the Wnt signaling pathway during the 2nd telogen was demonstrated by increased expression of *Axin2*, *Wnt7b* and *Wnt10b*, which explains the findings that a large portion of HFs were in the telogen–anagen transition state in hairpoor mice. In addition, we previously documented that another Wnt inhibitor, Dickkopf-related protein 1 (Dkk1), was overexpressed in hairpoor mouse [25]. Expression of Dkk1 in anagen and catagen HFs was demonstrated, and it is known to induce HF from anagen to catagen [46]. Taken together, these findings strongly suggest that the acceleration of HF cycling was led by aberrant Wnt signaling in the hairpoor mouse. This may be related to development of alopecia with aging in the hairpoor mouse, and further study is required in order to better elucidate this disorder. This study provided a basis for understanding the mechanism of hair loss in MUHH patients. Currently, there is no available treatment for hair loss induced by MUHH disease. If the reciprocal mechanism between HFSC and the *HR* gene in MUHH disease is fully understood, modulation of the signaling pathways such as Wnt might provide a valuable therapeutic opportunity.

## Conclusions

In conclusion, in HR overexpressing hairpoor mice, the HFSC quiescence is not maintained due to decreased HFSC niche signals including *Fgf18, Bmp6*, and *Sfrp1*. This leads to the telogen-to-anagen transition of HFs at the telogen phase, and proliferation of HF occurs through activation of the Wnt signaling in the hairpoor mouse (Fig. 6h). Consequently, this causes abnormalities of HF cycling, which eventually leads to development of alopecia. Our results indicate that HR plays an important role in HFSC quiescence by regulating the signaling networks in HF. Our study elucidates the molecular genetic etiology of MUHH disease and may provide therapeutic directions.

## Supplementary Information

**Additional file 1**:** Table S1**. List of gene specific primers.

**Additional file 2**:** Figure S1**. Hematoxylin and eosin (H&E) stain of hairpoor mouse (*+/Hr*^*Hp*^) back skin. Hematoxylin and eosin (H&E) stain of back skin of mice at indicated day. +/HrHp; the hairpoor mouse, *+/+*; the wild type mouse. Scale bar = 200 μm. P; postnatal day.

**Additional file 3**:** Figure S2**. Immunofluorescence analyses of HFSC niche. (**a**) Immunofluorescence analyses of K6 (red) and K15 (green) in wild type and hairpoor mouse at the telogen phases. Nuclei were counterstained with DAPI. (**b**) Quantification of K6 positive cells in K15 positive bulge cell. n=3 per genotype. Scale bar =50μm.

**Additional file 4: ﻿Figure S3**. Expression of Fgf18, Bmp6 and Wnt molecules in hairpoor mouse. Expression level of each gene was determined by qRT-PCR analyses and normalized against that of Succinate Dehydrogenase Complex Flavoprotein Subunit A (*Sdha*). The relative expression level was determined using those of the wildtype levels as reference. (**a,b**) Expression level of *Fgf18 *and *Bmp6* of the epidermis at the 1st and 2nd telogen phase of the hairpoor mouse. (**c,d,e,f**) Expression level of Wnt signaling molecules (*Sfrp1*, *Wnt7b*, *Axin2*, and *Wnt10b*) in the telogen phased total skin of the hairpoor mouse . n = 4 per genotype. Data are mean ± SD. *P

## Data Availability

The dataset used and/or analyzed during the current study is available from the corresponding author upon reasonable request.

## Abbreviations

HFSC: Hair follicle stem cell
HF: Hair follicle
Hr: Hairless
MUHH: Marie-Unna hypotrichosis
LRC: Label retaining cell
BMP: Bone morphogenetic protein
K6: Keratin 6
BrdU: 5-Bromodeoxy-uridine
LEF1: Lymphoid enhancer-binding factor1
LHX2: LIM homeobox 2
SOX9: SRY-box 9
NFATc1: Nuclear factor of activated T cell 1
TCF4: Transcription factor 4
Dkk1: Dickkopf-related protein 1

## References

[1] Greco V, Chen T, Rendl M, Schober M, Pasolli HA, Stokes N, Dela Cruz-Racelis J, Fuchs E (2009). A two-step mechanism for stem cell activation during hair regeneration. Cell Stem Cell.

[2] Nowak JA, Polak L, Pasolli HA, Fuchs E (2008). Hair follicle stem cells are specified and function in early skin morphogenesis. Cell Stem Cell.

[3] Muller-Rover S, Handjiski B, van der Veen C, Eichmuller S, Foitzik K, McKay IA, Stenn KS, Paus R (2001). A comprehensive guide for the accurate classification of murine hair follicles in distinct hair cycle stages. J Invest Dermatol.

[4] Plikus MV (2012). New activators and inhibitors in the hair cycle clock: targeting stem cells’ state of competence. J Investig Dermatol.

[5] Choi YS, Zhang Y, Xu M, Yang Y, Ito M, Peng T, Cui Z, Nagy A, Hadjantonakis AK, Lang RA (2013). Distinct functions for Wnt/beta-catenin in hair follicle stem cell proliferation and survival and interfollicular epidermal homeostasis. Cell Stem Cell.

[6] Lien W-H, Fuchs E (2014). Wnt some lose some: transcriptional governance of stem cells by Wnt/β-catenin signaling. Genes Dev.

[7] Kandyba E, Kobielak K (2014). wnt7b is an important intrinsic regulator of hair follicle stem cell homeostasis and hair follicle cycling. Stem Cells.

[8] Shen Q, Yu W, Fang Y, Yao M, Yang P (2017). Beta-catenin can induce hair follicle stem cell differentiation into transit-amplifying cells through c-myc activation. Tissue Cell.

[9] Huelsken J, Vogel R, Erdmann B, Cotsarelis G, Birchmeier W (2001). β-catenin controls hair follicle morphogenesis and stem cell differentiation in the skin. Cell.

[10] Hsu Y-C, Li L, Fuchs E (2014). Emerging interactions between skin stem cells and their niches. Nat Med.

[11] Kobielak K, Stokes N, de la Cruz J, Polak L, Fuchs E (2007). Loss of a quiescent niche but not follicle stem cells in the absence of bone morphogenetic protein signaling. Proc Natl Acad Sci.

[12] Hsu Y-C, Pasolli HA, Fuchs E (2011). Dynamics between stem cells, niche, and progeny in the hair follicle. Cell.

[13] Kimura-Ueki M, Oda Y, Oki J, Komi-Kuramochi A, Honda E, Asada M, Suzuki M, Imamura T (2012). Hair cycle resting phase is regulated by cyclic epithelial FGF18 signaling. J Investig Dermatol.

[14] Plikus MV, Mayer JA, de la Cruz D, Baker RE, Maini PK, Maxson R, Chuong C-M (2008). Cyclic dermal BMP signalling regulates stem cell activation during hair regeneration. Nature.

[15] Moraitis AN, Giguere V, Thompson CC (2002). Novel mechanism of nuclear receptor corepressor interaction dictated by activation function 2 helix determinants. Mol Cell Biol.

[16] Potter GB, Beaudoin GMJ, DeRenzo CL, Zarach JM, Chen SH, Thompson CC (2001). The hairless gene mutated in congenital hair loss disorders encodes a novel nuclear receptor corepressor. Genes Dev.

[17] Hsieh JC, Sisk JM, Jurutka PW, Haussler CA, Slater SA, Haussler MR, Thompson CC (2003). Physical and functional interaction between the vitamin D receptor and hairless corepressor, two proteins required for hair cycling. J Biol Chem.

[18] Panteleyev AA, Paus R, Christiano AM (2000). Patterns of hairless (hr) gene expression in mouse hair follicle morphogenesis and cycling. Am J Pathol.

[19] Thompson CC, Sisk JM, Beaudoin GM (2006). Hairless and Wnt signaling: allies in epithelial stem cell differentiation. Cell Cycle.

[20] Ahmad W, Zlotogorski A, Panteleyev AA, Lam H, Ahmad M, Faiyaz ul Haque M, Abdallah HM, Dragan L, Christiano AM (1999). Genomic organization of the human hairless gene (HR) and identification of a mutation underlying congenital atrichia in an Arab Palestinian family. Genomics.

[21] Nam Y, Kim JK, Cha DS, Cho JW, Cho KH, Yoon S, Yoon JB, Oh YS, Suh JG, Han SS (2006). A novel missense mutation in the mouse hairless gene causes irreversible hair loss: genetic and molecular analyses of Hr m1Enu. Genomics.

[22] Wen Y, Liu Y, Xu Y, Zhao Y, Hua R, Wang K, Sun M, Li Y, Yang S, Zhang XJ (2009). Loss-of-function mutations of an inhibitory upstream ORF in the human hairless transcript cause Marie Unna hereditary hypotrichosis. Nat Genet.

[23] Baek IC, Kim JK, Cho KH, Cha DS, Cho JW, Park JK, Song CW, Yoon SK (2009). A novel mutation in Hr causes abnormal hair follicle morphogenesis in hairpoor mouse, an animal model for Marie Unna Hereditary Hypotrichosis. Mamm Genome.

[24] Kim JK, Kim E, Baek IC, Kim BK, Cho AR, Kim TY, Song CW, Seong JK, Yoon JB, Stenn KS (2010). Overexpression of Hr links excessive induction of Wnt signaling to Marie Unna hereditary hypotrichosis. Hum Mol Genet.

[25] Kim BK, Lee HY, Kim I, Choi K, Park J, Yoon SK (2014). Increased expression of Dkk1 by HR is associated with alteration of hair cycle in hairpoor mice. J Dermatol Sci.

[26] Braun KM, Watt FM (2004). Epidermal label-retaining cells: background and recent applications. J Investig Dermatol Symp Proc.

[27] Jensen KB, Driskell RR, Watt FM (2010). Assaying proliferation and differentiation capacity of stem cells using disaggregated adult mouse epidermis. Nat Protoc.

[28] Trempus CS, Morris RJ, Bortner CD, Cotsarelis G, Faircloth RS, Reece JM, Tennant RW (2003). Enrichment for living murine keratinocytes from the hair follicle bulge with the cell surface marker CD34. J Invest Dermatol.

[29] Plikus MV, Gay DL, Treffeisen E, Wang A, Supapannachart RJ, Cotsarelis G (2012). Epithelial stem cells and implications for wound repair. Semin Cell Dev Biol.

[30] Rhee H, Polak L, Fuchs E (2006). Lhx2 maintains stem cell character in hair follicles. Science.

[31] Horsley V, Aliprantis AO, Polak L, Glimcher LH, Fuchs E (2008). NFATc1 balances quiescence and proliferation of skin stem cells. Cell.

[32] Vidal VPI, Chaboissier M-C, Lützkendorf S, Cotsarelis G, Mill P, Hui C-C, Ortonne N, Ortonne J-P, Schedl A (2005). Sox9 Is essential for outer root sheath differentiation and the formation of the hair stem cell compartment. Curr Biol.

[33] Lien W-H, Polak L, Lin M, Lay K, Zheng D, Fuchs E (2014). In vivo transcriptional governance of hair follicle stem cells by canonical Wnt regulators. Nat Cell Biol.

[34] Beaudoin GM, Sisk JM, Coulombe PA, Thompson CC (2005). Hairless triggers reactivation of hair growth by promoting Wnt signaling. Proc Natl Acad Sci U S A.

[35] Mardaryev AN, Meier N, Poterlowicz K, Sharov AA, Sharova TY, Ahmed MI, Rapisarda V, Lewis C, Fessing MY, Ruenger TM (2011). Lhx2 differentially regulates Sox9, Tcf4 and Lgr5 in hair follicle stem cells to promote epidermal regeneration after injury. Development.

[36] Kadaja M, Keyes BE, Lin M, Pasolli HA, Genander M, Polak L, Stokes N, Zheng D, Fuchs E (2014). SOX9: a stem cell transcriptional regulator of secreted niche signaling factors. Genes Dev.

[37] Blanpain C, Lowry WE, Geoghegan A, Polak L, Fuchs E (2004). Self-renewal, multipotency, and the existence of two cell populations within an epithelial stem cell niche. Cell.

[38] Trempus CS, Morris RJ, Ehinger M, Elmore A, Bortner CD, Ito M, Cotsarelis G, Nijhof JGW, Peckham J, Flagler N (2007). cd34 expression by hair follicle stem cells is required for skin tumor development in mice. Can Res.

[39] Yang L, Wang L, Yang X (2008). Disruption of Smad4 in mouse epidermis leads to depletion of follicle stem cells. Mol Biol Cell.

[40] Bhattacharya S, Wheeler H, Leid M, Ganguli-Indra G, Indra AK (2015). Transcription factor CTIP2 maintains hair follicle stem cell pool and contributes to altered expression of LHX2 and NFATC1. J Investig Dermatol.

[41] Osada S-I, Minematsu N, Oda F, Akimoto K, Kawana S, Ohno S (2015). Atypical protein Kinase C Isoform, aPKCλ, is essential for maintaining hair follicle stem cell Quiescence. J Investig Dermatol.

[42] Peters F, Vorhagen S, Brodesser S, Jakobshagen K, Brüning JC, Niessen CM, Krönke M (2015). Ceramide synthase 4 regulates stem cell homeostasis and hair follicle cycling. J Investig Dermatol.

[43] Leishman E, Howard JM, Garcia GE, Miao Q, Ku AT, Dekker JD, Tucker H, Nguyen H (2013). Foxp1 maintains hair follicle stem cell quiescence through regulation of Fgf18. Development.

[44] Lim X, Tan SH, Yu KL, Lim SB, Nusse R (2016). Axin2 marks quiescent hair follicle bulge stem cells that are maintained by autocrine Wnt/beta-catenin signaling. Proc Natl Acad Sci U S A.

[45] Kim B-K, Baek I-C, Lee H-Y, Kim J-K, Song H-H, Yoon SK (2010). Gene expression profile of the skin in the 'hairpoor' (HrHp) mice by microarray analysis. BMC Genomics.

[46] Kwack MH, Kim MK, Kim JC, Sung YK (2012). Dickkopf 1 Promotes Regression of Hair Follicles. J Investig Dermatol.

