# Supplementary material for: The stem cell quiescence and niche signaling is disturbed in the hair follicle of the hairpoor mouse, an MUHH model mouse
Source: Stem Cell Res Ther. 2022 May 26;13:211. doi: 10.1186/s13287-022-02898-w (PMC9137081; doi:10.1186/s13287-022-02898-w)
Supplement: Supplementary file 1 — Additional file 1: Table S1. List of gene specific primers. [file 13287_2022_2898_MOESM1_ESM.docx]

| **Table S1. List of gene specific primers** | | |
| --- | --- | --- |
| **Genes** | **Accession Number** | **Sequences (5'-3')** |
| CD34 | NM_001111059 | F: caccgagccatatgcttaca |
|  |  | R: agcagacactagcaccagca |
| K6 | NM_008476 | F: aggtcaccatcaaccagagc |
|  |  | R: cttggtgtccaggaccttgt |
| Bmp6 | NM_007556 | F: ttcttcaaggtgagcgaggt |
|  |  | R: tagttggcagcgtagccttt |
| Fgf18 | NM_008005 | F: cctgcacttgcctgtgttta |
|  |  | R: cccaggacttgaatgtgctt |
| Sfrp1 | NM_003012 | F: tcagaggccatcattgaaca |
|  |  | R: gcaggtactggctcttcacc |
| Axin2 | NM_015732 | F: aagccccatagtgcccaaag |
|  |  | R: gtcctgggtaaatgggtgag |
| Wnt7b | NM_001163633 | F: tccgagtagggagtcgagag |
|  |  | R: agaaaagtcgatgccgtagc |
| Wnt10b | NM_011718 | F: gaatgctgctccgccgaggg |
|  |  | R: gctcctccagcatgtcgaag |
| Lef1 | NM_001276402 | F: tgagagcgaatgtcgtagct |
|  |  | R: gaggtggcagtgactgtgtc |
| Lhx2 | NM_001290646 | F: atgcaagctcaacctggagt |
|  |  | R: tgtgcatgtgaagcagttga |
| Sox9 | NM_011448 | F: cgagcactctgggcaatc |
|  |  | R: ggctcagctgctccgtct |
| Nfatc1 | NM_001164109 | F: aacgccctgctgaccaccgatagcact |
|  |  | R: cccgggtgccttccgtctcata |
| Tcf4 | NM_001083967 | F: gggaggaagagaaggtgtcc |
|  |  | R: aaacggggttaaggagcagt |
| Gapdh | NM_001289726 | F: aactttggcattgtggaagg |
|  |  | R: acacattgggggtaggaaca |
| Sdha | NM_023281 | F: agaaaggccaaatgcagctc |
|  |  | R: gtgagaacaagaaggcatcagc |
