# Supplementary figures and images for: The stem cell quiescence and niche signaling is disturbed in the hair follicle of the hairpoor mouse, an MUHH model mouse
Source: Stem Cell Res Ther. 2022 May 26;13:211. doi: 10.1186/s13287-022-02898-w (PMC9137081; doi:10.1186/s13287-022-02898-w)

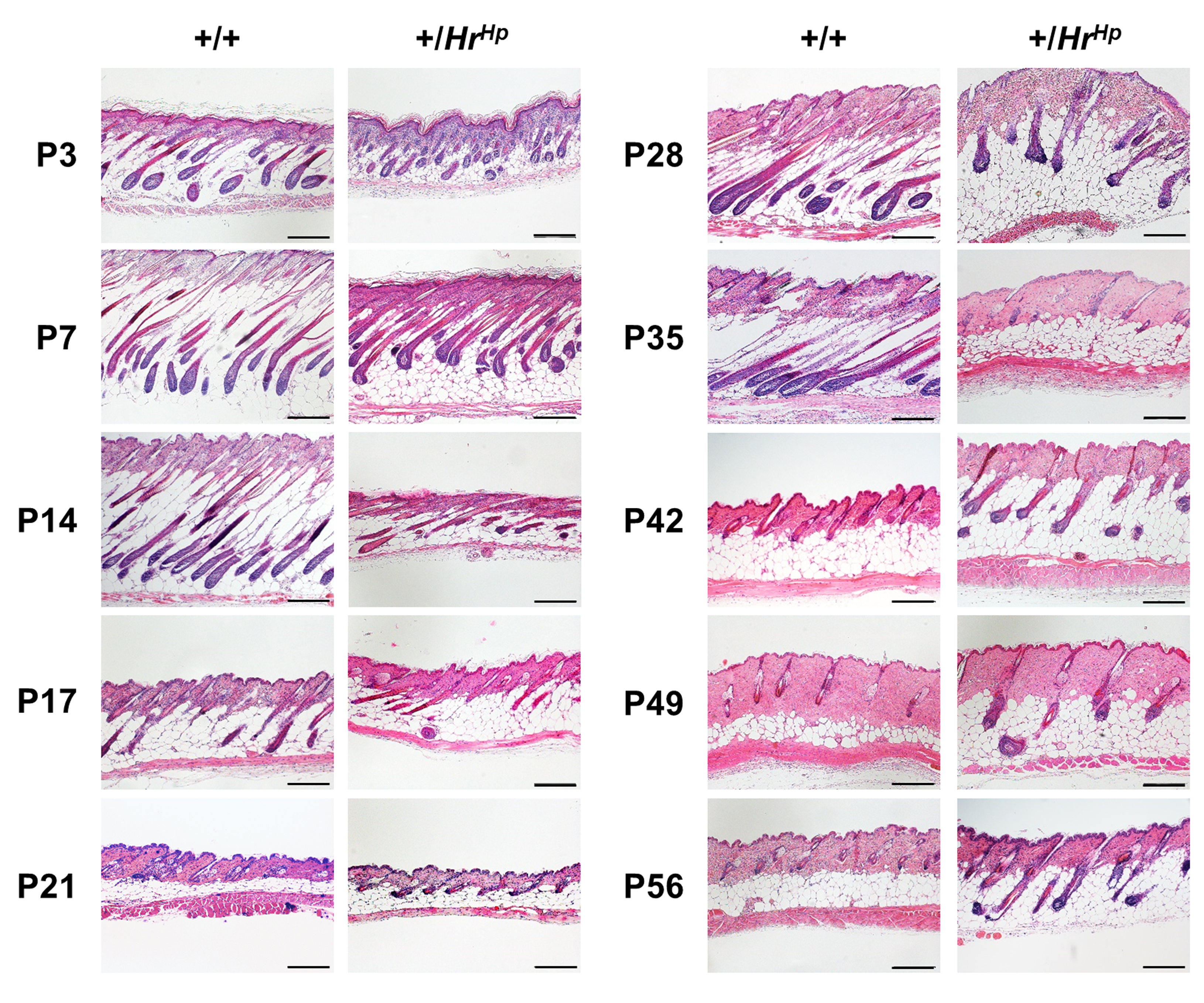

Supplement: Supplementary file 2 — Additional file 2: Figure S1. Hematoxylin and eosin (H&E) stain of hairpoor mouse (+/HrHp) back skin. Hematoxylin and eosin (H&E) stain of back skin of mice at indicated day. +/HrHp; the hairpoor mouse, +/+; the wild type mouse. Scale bar = 200 μm. P; postnatal day. [file 13287_2022_2898_MOESM2_ESM.tif]

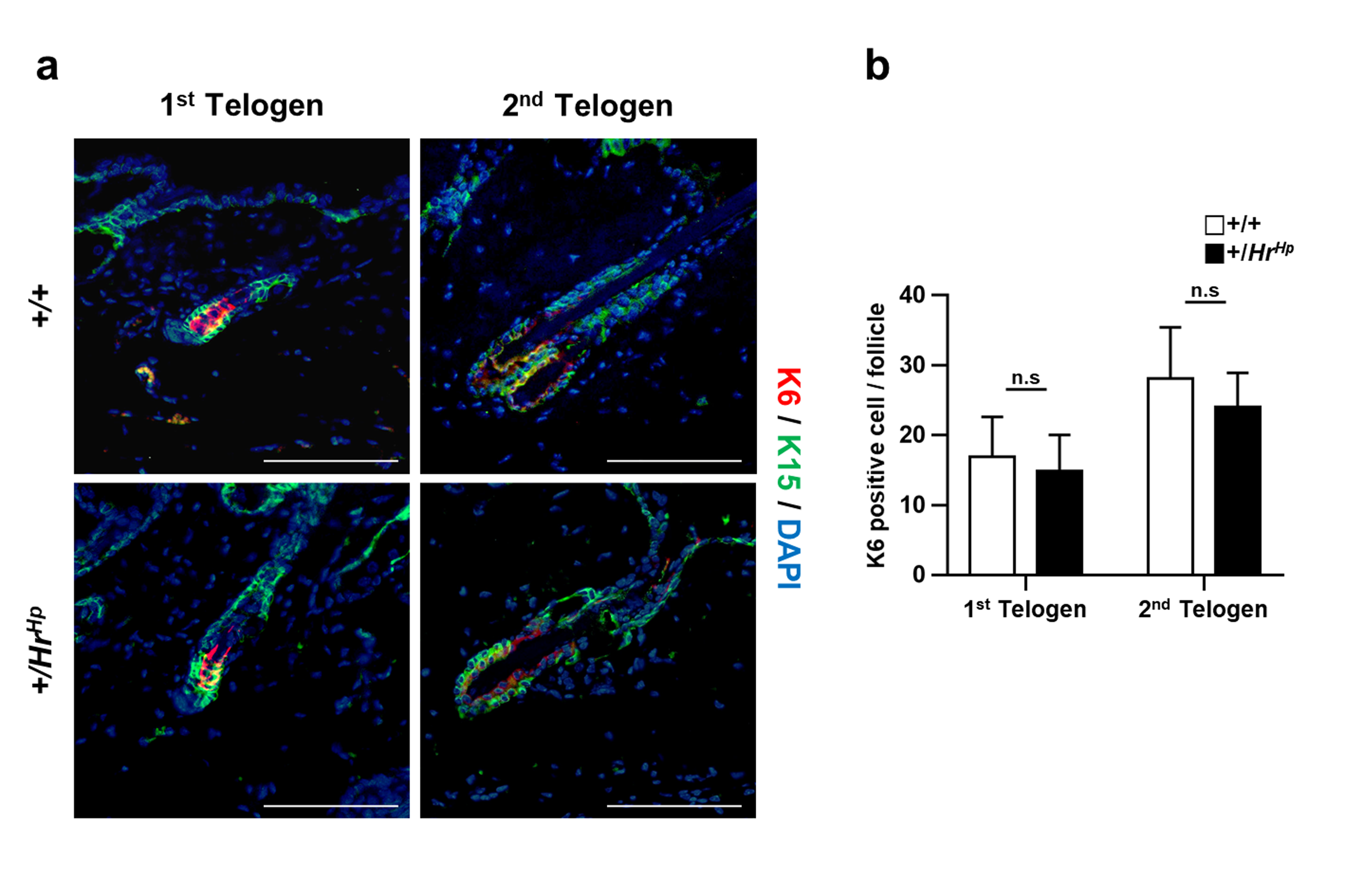

Supplement: Supplementary file 3 — Additional file 3: Figure S2. Immunofluorescence analyses of HFSC niche. (a) Immunofluorescence analyses of K6 (red) and K15 (green) in wild type and hairpoor mouse at the telogen phases. Nuclei were counterstained with DAPI. (b) Quantification of K6 positive cells in K15 positive bulge cell. n=3 per genotype. Scale bar =50μm. [file 13287_2022_2898_MOESM3_ESM.tif]

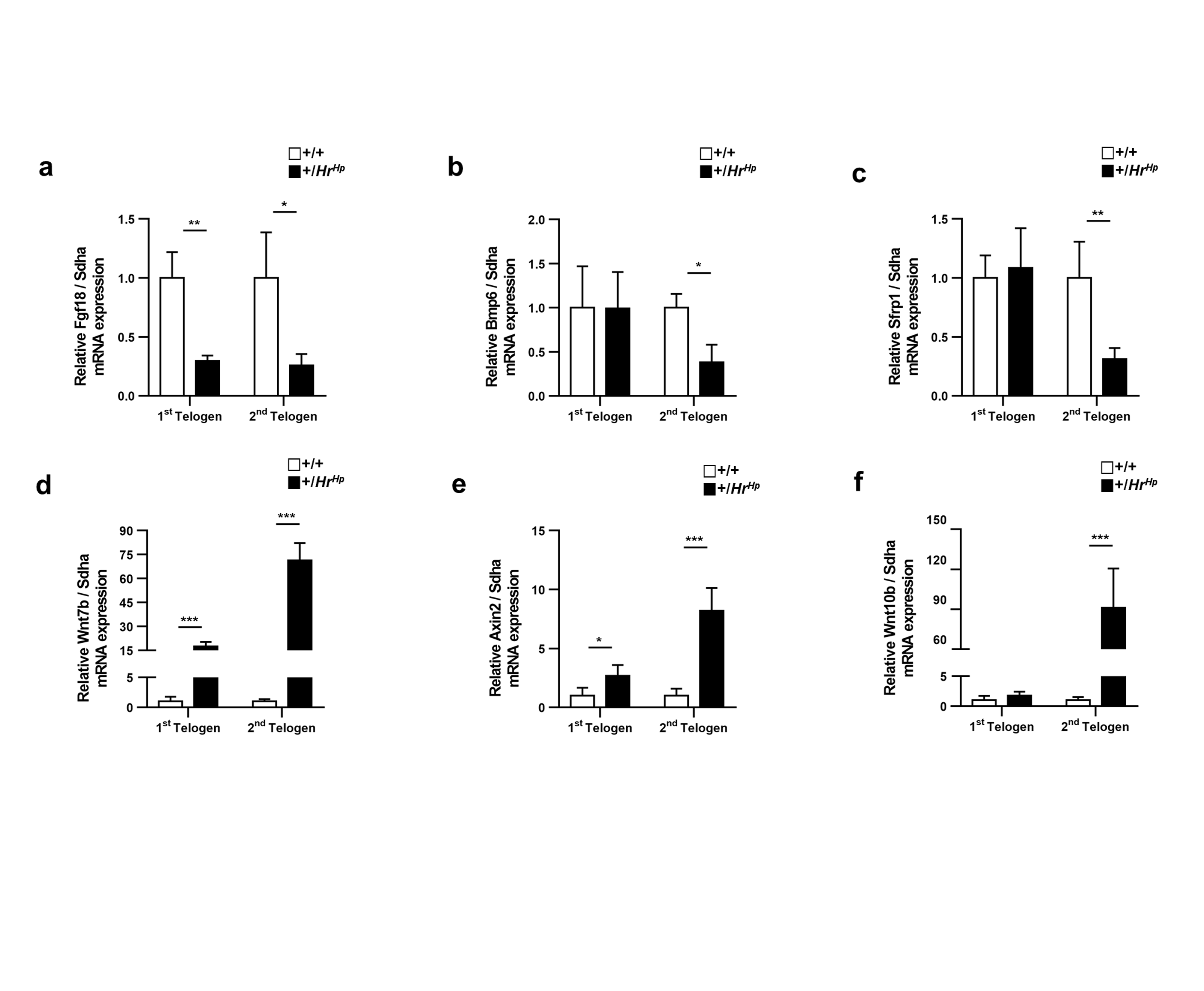

Supplement: Supplementary file 4 — Additional file 4: ﻿Figure S3. Expression of Fgf18, Bmp6 and Wnt molecules in hairpoor mouse. Expression level of each gene was determined by qRT-PCR analyses and normalized against that of Succinate Dehydrogenase Complex Flavoprotein Subunit A (Sdha). The relative expression level was determined using those of the wildtype levels as reference. (a,b) Expression level of Fgf18 and Bmp6 of the epidermis at the 1st and 2nd telogen phase of the hairpoor mouse. (c,d,e,f) Expression level of Wnt signaling molecules (Sfrp1, Wnt7b, Axin2, and Wnt10b) in the telogen phased total skin of the hairpoor mouse . n = 4 per genotype. Data are mean ± SD. *P [file 13287_2022_2898_MOESM4_ESM.tif]
